# Ethnophytotherapy Practices for Wound Healing among Populations of District Haripur, KPK, Pakistan

**DOI:** 10.1155/2019/4591675

**Published:** 2019-07-14

**Authors:** Zeeshan Siddique, Ghulam Mujtaba Shah, Hiwa M. Ahmed, Sobia Nisa, Abdullah Khan, Muhammad Idrees, Shumaila Naz, Syed Waqas Hassan, Muhammad Mohiuddin

**Affiliations:** ^1^Department of Biosciences, University of Wah, Wah Cantt, Pakistan; ^2^Department of Botany, Hazara University of Mansehra, Pakistan; ^3^Sulaimani Polytechnic University, Slemani, Kurdistan Regional Government, Iraq; ^4^Newcasle Center for Natural Therapy, Slemani, Ranya, Kurdistan Region, Iraq; ^5^Department of Microbiology, University of Haripur, KPK, Pakistan; ^6^Department of Environmental Sciences, University of Haripur, KPK, Pakistan; ^7^Department of Environmental Sciences, COMSATS Institute of Information Technology, Abbottabad, Pakistan

## Abstract

Wounds, burns, cuts, and scarring may cause a serious problem for human health if left untreated, and medicinal plants are identified as potentially useful for wound healing. Therefore, the study focused on ethnophytotherapy practices for wound healing from an unexplored area, Pakistan. Ethnophytotherapeutic information was collected through well-planned questionnaire and interview methods by targeting 80 informants (70 males and 10 females), in the study area. Data was analyzed through quantitative tools like use value (UV) and credibility level (CL). A total of forty wound healing plant species, belonging to twenty-nine families, were being used in forty-six recipes. Herbs constitute (35%), shrubs (30%), trees (30%), and climbers (5%) in the treatment of multiple human injuries. For remedies preparations, leaves were most frequently utilized (52%) followed by whole plant, flowers, twigs, roots, bulb, bark, rhizome, resin, oil, leaf gel, latex, gum, and creeper. The most form of herbal preparation was powder (34.7%) and poultice (32.6%), followed by decoction, bandaged and crushed, in which 40% internally and 60 % externally applied. The drugs from these plants seem to be widely used to cure wounds:* Acacia modesta*,* Aloe barbadensis*,* Azadirachta indica*,* Ficus benghalensis*,* Nerium oleander,* and* Olea ferruginea* with higher use values (0.75). Local people are still connected with ethnophytotherapies practices for curing wounds for several reasons. This ethnomedicine and the wound healing plants are under severe threats; thus conservation must be considered. Further research should be directed towards implementing pharmacological activity on these invaluable botanical drugs.

## 1. Introduction

Plants as medicine play an important role in the public health sector across the world. Patients have been utilizing medicinal plants for a long time to fulfill different daily needs and to maintain well-being [1]. Plants provide people with food, medicines, and fodder for livestock, as well as materials for construction of houses [2]. The history of discovery and use of different medicinal plants is as old as the history of discovery and use of plants for food [3]. From the history, it was revealed that the ancient people used herbal medicine for treatment of various diseases including burn injury, due to its simplicity, low cost, and affordable basis [4]. Today, the injury is a serious health problem across the world, often associated with high-costs and inefficient therapies [5] and affect people both physically and psychologically [6]. It is estimated that several million patients suffer from wounds, burns, and cuts every year, which may result in death when they did not get treated properly [7, 8]. Wound infections and related diseases are very common in developing and some developed countries due to unhygienic conditions [9, 10]. Wounds can be referred to as physical disabilities [11] and are marked as injury to normal skin structural, anatomical, physiological, and functional variation [12]. World Health Organization (WHO) 2018 data estimated that 180 000 people died every year due to burn injuries, and the vast majority of these deaths occur in low- and middle-income countries. Many medical plants support natural repairing process of skin [13, 14]. As described in the different literature, 70% of the wound healing drugs are of plant origin, 20% of mineral origin, and the remaining 10% consisting of animal products [15].

So, by keeping in view the importance of phytotherapies for wound healing, our study was conducted with the aims (i) to unveil the valuable wounds healing plants from District Haripur, KPK, Pakistan, as previously this area was unexplored in this regard, (ii) to record traditional folk knowledge and phytotherapies being used in wound healing practices, (iii) to record wound healing medicinal plants with highest use value (UV) and credibility level (CL) for further in vitro investigations, (iv) to identify potential threats, and (v) to provide baseline data for phytochemists, pharmacologists, and conservationists for further future study.

## 2. Methods

### 2.1. Study Area

**Figure 1:**
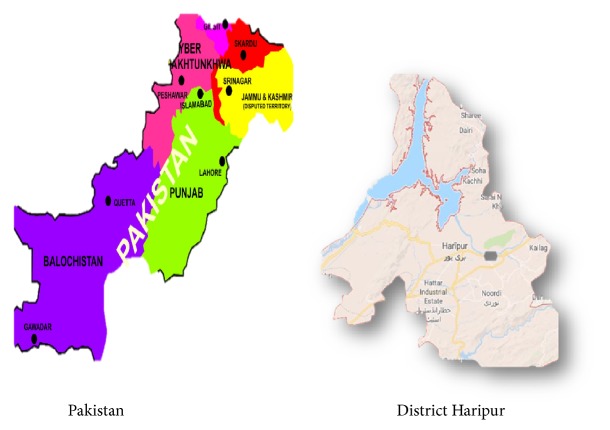
Map of study area in Pakistan.

Haripur district is situated in Hazara division of Khyber Pakhtunkhwa province of Pakistan (Figure 1) at latitude 33°-44′ to 34°-22′ and longitude 72°-35′ to 73°-15′ and about 610 meters above the sea level. A total area of the district is 1725 square kilometers. Haripur was founded in 1822 by Hari singh Nalva, a Sikh General of Ranjit Singh's army. He was the Governor of Kashmir in 1822-23 A.D. after whom it is named. District Haripur has three tehsils, namely, Khanpur, Ghazi, and Haripur. The whole district is subdivided into 45 Union Councils (UCs). According to National Institute of Population Studies (NIPS), the estimated population of the district was 857,000, in 2008, having population density of 497 persons per square kilometer. The district is predominantly a rural district where only 12% population lives in urban areas. Agriculture is the main source of livelihood of rural population of the district. Haripur district has distinct geographical significance as its boundaries touch Districts Mansehra, Abbottabad, Torghar, Buner, Swabi, Attock, Rawalpindi, and Federal capital Islamabad [16].

### 2.2. Field Survey

The whole study area was frequently visited and the main target sites in the study area were Khanpur, Garmthun, Najafpur, Dartian, Babotri, Baghpur dehri, Kohala, Nilan Bhoto, Jabri, Hattar, Kotnajibullah, Khalabat, Beer, Ghazi, Nara Amazai, etc. A field survey was aimed to collect field data and activities like (i) recording folk knowledge and phytotherapies being used in wound healing practices, (ii) plant's collection, (iii) local information about plants, (iv) identification of potential threats, (v) photography, etc. This survey was completed through well-planned questionnaires, interviews, and keen observations. Questionnaire method was also helpful in the documentation of folk indigenous knowledge. The interviews were helpful in investigation of local people and knowledgeable persons (farmers and herdsmen), who are mainly connected with plants and involved in traditional health care. During the field visits, total of 80 informants were approached randomly for questionnaires and interviews. Their description with respect to age, education, profession, etc. is given in (Table 1).

### 2.3. Plant Collection and Identification

The plant specimens collected were authenticated using the international plant name index (http://www.ipni.org), the plant list (www.theplantlist.org), and GRIN taxonomy site (http://www.ars-grin.gov/cgi-bin/npgs/html/queries.pl). The life form of investigated plants was categorized into herbs, shrubs, grasses, and trees (annual, biennial, or perennial), according to the system modified by Brown [17]. The collected plant specimens were identified by Prof. Dr. Ghulam Mujtaba Shah (Plant Taxonomist), Hazara University Mansehra (Pakistan), and by using Flora of West Pakistan [18] and Flora of Punjab [19]. The voucher specimens were deposited in the Herbarium, Department of Botany, Hazara University, Mansehra (Pakistan).

### 2.4. Identification of Potential Threats

During field survey, potential threats were identified through well-planned questionnaires, interviews, and keen observations. Therefore, the data obtained were tabulated and supported by photography. The main aim of the photography was to support field data with evidence. Photography of study area, as well as important plants and potential threats, was taken.

### 2.5. Statistical Analysis

The ethnophytotherapeutic data was statistically analyzed using Microsoft Office Excel software (2010). The use value (UV) of plant species were also determined [1, 20].

Use value (UV) determines the relative importance of uses of plant species. It is calculated using the following formula:

(1) $$\begin{array}{c} {UV}_{i}=\frac{ƩUi}{Ni} \end{array}$$

where “UV” indicates use value of individual species, “U” is the number of uses recoded for that species, and “N” represents the number of informants who reported that species.

Credibility level (CL) of plant species: to determine credibility level of plants, a simple statistical tool was developed given name “credibility level” (CL). First of all, those plants are chosen which were reported by more than five informants with the same use. Then each plant was evaluated under the following formula:

(2) $$\begin{array}{c} CL=\frac{Ni}{ƩN_{i}}\times 100 \end{array}$$

where “CL” indicates credibility level of plant species, “N” is the number of informants recorded for this plant, and “*Ʃ*N” represents the total number of informants for all plant species. CL value of each plant was tabulated in ascending series. The higher CL value is the most credibility of the medical plant used as ethnomedicine among communities in the study area.

## 3. Results

**Figure 2:**
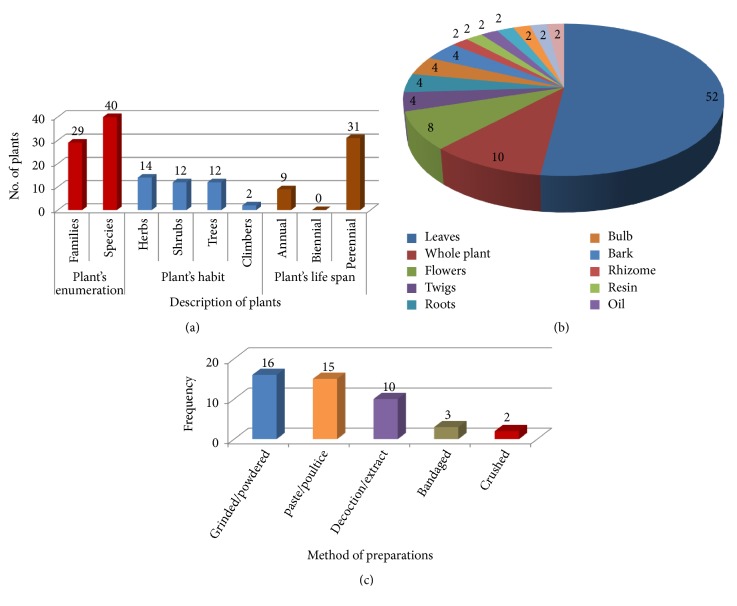
(a) Description of medicinal plants; (b) type of botanical parts; (c) methods of herbal drug preparation used for wound healing in the study area.

During the present study, data on 40 medicinal plant species belonging to 29 families that are being used in wound healing phytotherapies were collected from the study area. From those, herbs (35%) were the most widely used by local patients to treat various injury problems in the study areas of Haripur, followed by shrubs (30%), trees (30%), and climber species (5%) in which there were (22.5%) annual and (77.5%) perennial plant species as shown in Figure 2(a). Detailed information about each plant pertaining to the botanical name, voucher number, local name, family, habit, lifespan, locality, plant parts used, ethnophytotherapies, number of informants, etc. is listed in Table 2. People of District Haripur use different parts of the plants for wound healing phytotherapies as shown in Figure 2(b). Among those plant parts, leaves were the most frequently used (52%) followed by whole plant (10%), flowers (8%), twigs, roots, bulb and bark (4% each) and rhizome, resin, oil, leaf gel, latex, gum, and creeper (2% each).

The methods of preparation for 46 recorded recipes fall into 5 categories, viz., plant parts used as decoction/extract (21.7%), Grinded/powdered form (34.7%), paste/poultice (32.6%), crushed (4.3%), and bandaged (6.5%) as shown in Figure 2(c).

Study also revealed that local people use wound healing phytotherapies both internally and externally. These treatments involve 40% internal and 60 % external use. Use value (UV) of plant species was also calculated in order to determine the relative importance of plant in study area with respect to usage. Results showed that* Acacia modesta*,* Aloe barbadensis*,* Azadirachta indica*,* Ficus benghalensis*,* Nerium oleander,* and* Olea ferruginea* have higher use values (UVs), i.e., 0.75, while species* Solanum surattense*,* Punica granatum*,* Mentha arvensis,* and* Allium sativum* have lower use values (UVs), i.e., 0.07, 0.11, 0.12, and 0.12, respectively, as shown in Table 2. CL value was also calculated to check the credibility of plants used in study area. CL values obtained are given in Table 3. The higher CL value is the most credibility of plant used as ethnomedicine among District Haripur communities. Results showed that 20 plants in study area are more credible in efficacy than others. Of these the highest CL value was recorded for* Dodonaea viscosa,* i.e., 16.25; hence it is the most credible, in contrast to* Ziziphus nummularia* which showed the lowest CL value i.e., 6.25, among these 20 plant species in study area.

During the study, it was also explored that plant resources of District Haripur are under severe threats. The major threats identified were subsequent forest fires during the summer, over grazing (normal and nomadic), overexploitation, mining activities, etc. as shown in Table 4. The important indigenous knowledge of plants is being confined to the older people mostly, as the young generations has little interest in such traditional practices and mainly because of transforming lifestyle and culture among the youth. This can be deduced from the informant's description by age showing that there were only 6.2% informants below 30 years of age.

## 4. Discussion

Since time immemorial, people have been used various parts of plants and its extracts in the treatment and prevention of many ailments [21]. This knowledge varies from region to region and from the community to community according to the presence of herbs and their historical uses [22]. In similar investigates, many other researchers [23–26] have significantly explored wound healing medicinal plants from other parts of the world. This is also confirmed by several other studies showing that herbs and plant organ leaves could be a major herbal remedy considered for many human ailments around the world including wounds and burns [27]. Many of these herbal medicines are used for wound healing in the form of (teas, decoctions, tinctures, syrups, oils, ointments, poultices, and infusions), as a safe and reliable natural substance derived from medicinal herbs [28].

Comparative analysis shows that ethnophytotherapies for wound healings in District Haripur may or may not be similar in use with the other published literature reported from various other parts of the world. For example, present use of leaf juice of* Melia azedarach *is used to cure pimples and burns, while Ahmad [29] reported that this plant is beneficial against scabies, carbuncles, and abscess in district Attock. In Indian traditional medicine, species of the following genera are commonly used to treat wound and related injuries;* Abutilon*,* Achyranthes*,* Acorus*,* Aegle*,* Aerva*,* Aloe*,* Azadirachta*,* Bambusa*,* Bidens*,* Boerhaavia*,* Butea*,* Caesalpinia*,* Calotropis*,* Carissa*,* Cassia*,* Cucumis*,* Curcuma*,* Cynodon*,* Datura*,* Dodonaea*,* Eclipta*,* Euphorbia*,* Ficus*,* Hyptis*,* Lantana*,* Leucas*,* Morinda*,* Ocimum*,* Opuntia*,* Pavetta*,* Pergularia*,* Plumbago*,* Pongamia*,* Sida*,* Smilax*,* Terminalia*,* Tridax*,* Vitex,* and* Zizyphus *[30].* Pistacia atlantica* subsp.* kurdica* Zohary can be used externally to treat skin injury as well as internally for abdominal pain [31]. A review of the previously published literature shows that the most species display a range of pharmacological activities that are suggested to have wound healing potential. For example,* Acacia modesta* has anti-inflammatory properties [32].* Ocimum basilicum* holds antibacterial activity [33, 34].* Berberis lyceum* retains antifungal properties [35, 36]. In rats, methanolic extract ointment from leaves of* Adhatoda vasica* had significant wound healing activity compared to standard drug [37]. A study by Ahmed [1] showed the importance of* Allium sativum* for antidandruff, intestinal worms, blood circulation, rheumatism, stimulant, cancer, cholera, alopecia areata, tuberculosis, and plague in Kurdistan region of Iraq, while in our study the same species was used to treat wounds by insect. The same author reported* Althaea officinalis* to treat burns in the form of poultice, which is exactly similar to our results. This is due to that fact that there may be several factors playing a role in establishing knowledge of ethnobotany and ethnomedicine particularly in rural areas, such as phytogeographical and ecological characteristics, demographic and ethnic structure as well [28]. This comparative analysis strengthens the value of the wound healing knowledge from our study location for further future studies.

## 5. Conclusion

Plant extracts have been demonstrated as important potential herbal remedies in many traditional medicinal systems around the world for wound repair and tissue regeneration. People of District Haripur (study area) rely mainly on ethnophytotherapies for wound healing. This is because of traditional culture, easy availability, and cheaper source of herbal medicines. Local people have sufficient knowledge about wound healing practices and saved mostly by older informants. In the study area both the wound healing, medicinal plants, and folk traditional knowledge are getting depleted thus conservation is required to maintain this valuable natural resource. Further pharmacological studies are recommended to validate the current herbal traditional knowledge.

## Figures and Tables

**Table 1:** Demographic characteristics of participants.

*By age*	10-20 years	20-30 years	30-40 years	40-50 years	50-60 years	Above 60 years	%
Male	0	05	10	15	18	22	87.5 %
Female	---	---	---	03	04	03	12.5 %
*Total*	---	(6.2%)	(12.5%)	(22.5%)	(27.5%)	(31.2%)	---
*By qualification*	Illiterate	Primary	Middle	Secondary	Higher secondary	Higher education	%
Male	16	18	14	14	06	02	87.5 %
Female	05	03	02	---	---	---	12.5 %
*Total*	(26.2%)	(26.2%)	(20%)	(17.5%)	(7.5%)	(2.5%)	---
*By profession*	Farmers	Herdsmen (nomadic)	Hakeem	Teachers	Shop-keeper	Laborers	%
Male	45	15	03	02	02	03	87.5 %
Female	08	02	---	---	---	---	12.5 %
*Total*	*(66.2*%)	*(21.2*%)	*(3.7*%)	*(2.5*%)	*(2.5*%)	*(3.7*%)	---

**Table 2:** Ethnophytotherapies for wounds healing in District Haripur.

No	Botanical Name/Voucher No.	Local Name	Family	Habit	Life span	Locality	Part Used	Ethno-Phytotherapies	No. of informants	U.V
1	*Acacia modesta *Wall. 01-Z	Phulai	Leguminosae	Tree	Perennial	Dartian	Twigs, bark & leaves	For gum bleedings: Twigs are used as *masvak*. For mouth boils: Decoction of bark is gargled several times a day. For abscesses, boils & adulthood poxes: Half spoon powdered leaves are taken with water for a few days.	04	0.75

2	*Acacia nilotica *(L.) Delile 02-Z	Kikar	Leguminosae	Tree	Perennial	Najafpur	Flowers, leaves & gum	Formouth ulcer: $1/4^{{th}^{}}$ spoon of powdered flowers and leaves are taken with water in morning and evening. For stomach ulcer: 1/4^th^ spoon of powdered gum is taken with milk or water.	04	0.5

3	*Achyranthes aspera* L. 03-Z	Puth-Kanda, Lehndi booti	Amaranthaceae	Herb	Perennial	Khanpur	Leaves	For wound washing & healing: Decoction of leaves is used for washing the wounds and then poultice of leaves is applied.	05	0.4

4	*Adhatoda vasica *Nees 05-Z	Bhaikur, aroosa	Acanthaceae	Shrub	Perennial	Halli	Leaves	For pimples: 1/2 cup of leaves juice is taken 2 times a day. For skin wounds: Powdered leaves are applied topically.	08	0.25

5	*Allium cepa *L. 08-Z	Payaz	Amaryllidaceae	Herb	Perrenial	Khanpur	Bulb	For wound healing: Inner bulb scale is heated in mustard oil and bandaged on wounds.	02	0.5

6	*Allium sativum *L. 09-Z	Thoom	Amaryllidaceae	Herb	Annual	Khanpur	Bulb	For animal and insect bites: Grinded in vinegar and made into a paste and applied.	08	0.12

7	*Aloe vera *(L.) Burm.f. 10-Z	Kanwar-ghandal	Xanthorrhoeaceae	Herb	Perennial	Dara	Leaf gel	For burn, wounds and other skin problems: Gel of leaves is burnt over the fry pan and applied topically.	04	0.75

8	*Althaea officinalis *L. 11-Z	Khatmi	Malvaceae	Herb	Annual	Halli	Flowers & leaves	For skin burns: Powdered flowers and leaves are applied.	04	0.25

9	*Amaranthus viridis *L. 12-Z	Chaleray	Amaranthaceae	Herb	Annual	Jabri	Leaves	For healing of boils & abscesses: The upper surfaces of leaves are smeared with mustard oil, warmed gently and bandaged.	06	0.33

10	*Azadirachta indica *A.Juss. 13-Z	Nim	Meliaceae	Tree	Perennial	Bagla	Leaves	For skin pimples, boils & abscesses: Leaves are dipped in water at night. In morning one spoon of this water is taken.	04	0.75

11	*Berberis lycium* Royle14-Z	Simbulu	*Berberidaceae*	Shrub	Perennial	Dartian	Root	For mouth boils & internal wounds: 1/4^th^ spoon of dried powdered root is taken with water.	10	0.2

12	*Bombax ceiba *L. 15-Z	Sumbal	Malvaceae	Tree	Perennial	Daboola	Stem bark	For wound healing: Bark paste is applied topically.	02	0.5

13	*Cannabis sativa *L. 21-Z	Pang, bhang	Cannabaceae	Sub shrub	Annual	Hattar	Leaves	For wounds: Leaves are bound over the wounds.	02	0.5

14	*Cissampelos pareira *L. 22-Z	Phalaan jarhi, Ghora-sum	Menispermaceae	Climer	Perennial	Najafpur	Leaves	For wounds & itching: Crushed leaves are applied.	06	0.3

15	*Curcuma longa *L. 31-Z	Haldi	Zingiberaceae	Herb	Perennial	Khanpur	Rhizome	For internal wounds: One spoon of dried grounded rhizome is mixed in one cup of hot milk and taken at night.	07	0.14

16	*Cuscuta reflexa *Roxb.33-Z	Bail	*Convolvulaceae*	Climber	Perennial	Najafpur	Creepers	For pimples & wounds: Creeper is crushed after boiling and tied over.	05	0.4

17	*Cynodon dactylon *(L.) Pers. 40-Z	Khabal	Poaceae	Herb	Perennial	Nara Amazai	Whole plant	For nose and wound bleedings: Paste of whole plant is applied.	08	0.25

18	*Dodonaea viscosa* (L.) Jacq. 55-Z	Sanatha	Sapindaceae	Shrub	Perennial	Garam Thoon	Leaves	For skin boils & wounds: Powdered leaves are applied.	13	0.15

19	*Eucalyptus globulus* Labill. 58-Z	Gond	Myrtaceae	Tree	Perennial	Khanpur	Oil	For skin wounds & fungal infections: Massage of Eucalyptus oil is useful.	08	0.25

20	*Ficus benghalensis L. * 86-Z	Bohr	Moraceae	Tree	Perennial	Bandi	Leaves	For pimples, acne & rashes: Paste of leaves is applied.	04	0.75

21	*Ficus carica *L. 90-Z	Anjeer	Moraceae	Tree	Perennial	Daboola	Milky latex	For wounds healing: Milky latex is applied twice a day.	05	0.2

22	*Lantana camara *L. 106-Z	---	Verbenaceae	Shrub	Perennial	Hattar	Leaves & twigs	For wounds swelling: Decoction of leaves is applied.	02	0.5

23	*Lawsonia inermis *L. 109-Z	Mehendi	Lythraceae	Tree	Perennial	Sarhadna	Leaves	For wounds: Leaf is grounded into paste and applied to get relief from burning sensation.	02	0.5

24	*Malvastrum coromandelianum (*L.) Garcke111-Z	Dhamni boti	Malvaceae	Sub shrub	Annual or biennial	Najafpur	Leaves	For ringworms & wounds: Paste of leaves is applied.	03	0.66

25	*Melia azedarach *L.124-Z	Daraik, bakain	Meliaceae	Tree	Perennial	Sarhadna	Leaves	For skin pimples: 1/2 cup of leaves juice is taken in morning. For burns: Fresh leaf extract is applied externally.	11	0.18

26	*Mentha arvensis *L.130-Z	Podina	Lamiaceae	Herb	Perennial	Mankrai	Leaves	For insect bites wound: Leaves are grinded and bruised and applied topically.	08	0.12

27	*Morus nigra* L. 145-Z	Kala toot, she-toot	Moraceae	Tree	Perennial	Dara	Leaves	For snake bite: Leaf extract is applied.	03	0.33

28	*Nasturtium officinale* R.Br. 150-Z	Tara meera	Brassicaceae	Herb	Perennial	Khanpur	Leaves	For skin allergies & pimples: 1/4^th^ spoon of dried powdered leaves are taken with water daily.	03	0.66

29	*Nerium oleander *L.162-Z	Kundair	Apocynaceae	Shrub	Perennial	Najafpur	Leaves & roots	For scabies and skin swellings: Decoction of leaves is directly applied. For scorpion bite: Root paste is applied over the wound.	04	0.75

30	*Ocimum basilicum* L. 164-Z	Niaz-bo	Lamiaceae	Herb	Annual	Khanpur	Leaves	For insect bite wounds: powdered leaves are rubbed.	03	0.33

31	*Olea europaea subsp. cuspidata *(Wall. & G.Don) Cif. 170-Z	Kaho	Oleaceae	Tree	Perennial	Choi	Leaves & twigs	For skin boils and pimples: 1 cup of leaf tea is taken once in a day. For mouth boils: Young twigs are chewed under the teeth.	04	0.75

32	*Rydingia limbata *(Benth.) Scheen & V.A.Albert. 173-Z	Chita kanda, bamboli	Lamiaceae	Shrub	Perennial	Najafpur	Whole plant	For wounds: Whole plant is powdered, mixed in butter and applied.	02	0.5

33	*Oxalis corniculata *L. 178-Z	Khat-matra	Oxalidaceae	Herb	Annual	Halli	Leaves	For skin inflammations: Powdered leaves are applied as a poultice.	04	0.25

34	*Pinus roxburghii* Sarg. 180-Z	Chir	Pinaceae	Tree	Perennial	Muslimabad	Resin	For skin burns, boils & wounds: Resin is applied externally.	06	0.5

35	*Punica granatum *L. 195-Z	Daruna	Lythraceae	Shrub	Perennial	Barkote	Flowers	For gum bleedings: Dried powdered flowers are used as tooth powder.	09	0.11

36	*Rumex hastatus* D. Don 206-Z	Katmat, tehtur	Polygonaceae	Shrub	Perennial	Najafpur	Leaves	For wounds: Leaves paste is applied.	02	0.5

37	*Solanum americanum* Mill.	Kach mach	Solanaceae	Herb	Annual	Joulian	Whole plant	For wound and boils: Whole plant paste is applied externally as a poultice.	05	0.4

38	*Solanum* surattense Burm. f. 240-Z	Mohkree	Solanaceae	Herb	Annual	Khoi Kaman	Whole plant	For skin infection: Dried powdered plant is applied topically.	13	0.07

39	*Woodfordia fruticosa *(L.) Kurz. 256-Z	Taawi, dhawi	Lythraceae	Shrub	Perennial	Najafpur	Leaves	For skin wounds: Poultice of leaves is applied externally.	06	0.16

40	*Ziziphus nummularia *(Burm.f.) Wight & Arn.282-Z	Beri	Rhamnaceae	Shrub	Perennial	Sarahdna	Leaves	For wounds: Leaf paste is applied over the wounds. For mouth & gum bleedings: Leaves are boiled in water, and gargled several times a day.	05	0.4

**Table 3:** Credibility level (CL) value for herbal drugs.

No	Botanical Name	Local Name	Locality	Part Used	Used against	C.L Values
1	*Dodonaea viscosa* (L.) Jacq.	Sanatha	Garam Thoon	Leaves	Skin boils & wounds	16.25
2	*Solanum surattense* Burm. f.	Mohkree	Khoi Kaman	Whole plant	Skin infection	16.25
3	*Melia azedarach *L.	Daraik, bakain	Sarhadna	Leaves	Skin pimples & burns	13.75
4	*Berberis lycium *Royle	Simbulu	Dartian	Root	Mouth boils & internal wounds	12.5
5	*Punica granatum *L.	Daruna	Barkote	Flowers	Gum bleedings	11.25
6	*Adhatoda vasica *Nees	Bhaikur, aroosa	Halli	Leaves	For pimples & skin wounds	10
7	*Allium sativum *L	Thoom	Khanpur	Bulb	Animal and insect bites	10
8	*Cynodon dactylon *(L.) Pers.	Khabal	Nara Amazai	Whole plant	Nose & wound bleedings	10
9	*Eucalyptus globulus* Labill.	Gond	Khanpur	Oil	Skin wounds & fungal infections	10
10	*Mentha arvensis *L.	Podina	Mankrai	Leaves	Insect bites wound	10
11	*Curcuma longa *L.	Haldi	Khanpur	Rhizome	Internal wounds	8.75
12	*Amaranthus viridis *L.	Chaleray	Jabri	Leaves	Healing of boils & abscesses	7.5
13	*Cissampelos pareira *L.	Phalaan jarhi, Ghora-sum	Najafpur	Leaves	Wounds & itching	7.5
14	*Pinus roxburghii* Sarg.	Chir	Muslimabad	Resin	Skin burns, boils & wounds	7.5
15	*Woodfordia fruticosa *(L.) Kurz.	Taawi, dhawi	Najafpur	Leaves	Skin wounds	7.5
16	*Achyranthes aspera *L.	Puth-Kanda, Lehndi booti	Khanpur	Leaves	Wound washing & healing	6.25
17	*Cuscuta reflexa *Roxb	Bail	Najafpur	Creepers	Pimples & wounds	6.25
18	*Ficus carica *L.	Anjeer	Daboola	Milky latex	Wounds healing	6.25
19	*Solanum americanum* Mill.	Kach mach	Joulian	Whole plant	Wound and boils	6.25
20	*Ziziphus nummularia *(Burm.f.) Wight & Arn.	Beri	Sarahdna	Leaves	Wounds and mouth & gum bleedings	6.25

**Table 4:** Major threats to the wound healing plants in the study area.

Locality/Threat	Fire	Over grazing (nomadic)	Over exploitation (Cutting etc.)	Mining activities
Khanpur	+	+	-	+
Garmthun	+	+	+	+
Najafpur	+	+	+	+
Dartian	+	+	+	+
Babotri	+	+	+	-
Baghpur dehri	+	+	+	+
Kohala	+	+	-	+
Nilan Bhoto	-	+	+	-
Jabri	+	+	+	+
Hattar	+	+	-	-
Sarae Nehmat Khan	-	+	-	-
Pharala	-	+	+	+
Beer	+	+	+	-
Ghazi	-	+	-	-
Nara Amazai	-	+	+	-

## Data Availability

The data used to support the findings of this study are included within the article.
